# Receptor-Mediated Mitophagy Rescues Cancer Cells under Hypoxic Conditions

**DOI:** 10.3390/cancers13164027

**Published:** 2021-08-10

**Authors:** Alibek Abdrakhmanov, Maria A. Yapryntseva, Vitaliy O. Kaminskyy, Boris Zhivotovsky, Vladimir Gogvadze

**Affiliations:** 1 Faculty of Medicine, MV Lomonosov Moscow State University, 119991 Moscow, Russia; alibek.abdrakhmanov@gmi.oeaw.ac.at (A.A.); smariaal@mail.ru (M.A.Y.); Boris.Zhivotovsky@ki.se (B.Z.); 2 Institute of Environmental Medicine, Division of Toxicology, Karolinska Institutet, Box 210, SE-171 77 Stockholm, Sweden; Vitaly.Kaminsky@ki.se

**Keywords:** cancer, hypoxia, cell death, autophagy, mitophagy, mitochondria

## Abstract

**Simple Summary:**

Stimulation of cell death is a promising strategy for tumor cell elimination. Many tumors develop under hypoxic conditions, which alter tumor cell biology and makes them resistant to cell death stimuli. One of the mechanisms responsible for tumor cell resistance to treatment could be the stimulation of mitophagy (mitochondrial quality control), which eliminates mitochondria that can trigger the mitochondrial pathway in apoptosis. We found that hypoxia stimulates mitophagy via enhanced expression of BNIP3 and BNIP3L proteins, which are involved in receptor-mediated mitophagy.

**Abstract:**

Targeting mitochondria with thenoyltrifluoroacetone (TTFA), an inhibitor of Complex II in the respiratory chain, stimulated cisplatin-induced apoptosis in various cell lines in normoxia but not in hypoxia. This can be explained by the elimination of mitochondria involved in triggering apoptotic cell death by mitophagy, either Parkin-dependent or receptor-mediated. Treatment with TTFA alone or in combination with cisplatin did not cause accumulation of PINK1, meaning that under hypoxic conditions cells survive through activation of a receptor-mediated pathway. Hypoxia triggers the accumulation of BNIP3 and BNIP3L (also known as NIX), key participants in receptor-mediated mitophagy. Under hypoxic conditions, stimulation of autophagy, as assessed by the accumulation of lipidated form of LC3 (LC3II), was observed. To exclude the contribution of canonical macroautophagy in LC3II accumulation, experiments were performed using U1810 cells lacking ATG13, a key enzyme of macroautophagy. Despite the absence of ATG13, hypoxia-mediated accumulation of LC3II was not affected, underlying the importance of the receptor-mediated pathway. In order to prove the protective role of BNIP3 against cisplatin-induced apoptosis, BNIP3-deficient A549 cells were used. Surprisingly, a BNIP3 knockout did not abolish hypoxia-induced protection; however, in cells lacking BNIP3, a compensatory upregulation of BNIP3L was detected. Thus, in the absence of BNIP3, mitophagy could be maintained by BNIP3L and lead to cell death suppression due to the elimination of proapoptotic mitochondria. When both BNIP3 and BNIP3L were knocked out, the inhibitory effect of hypoxia on apoptosis was diminished, although not abolished completely. Undoubtedly, receptor-mediated mitophagy is likely to be one of the mechanisms responsible for cell death suppression under hypoxic conditions.

## 1. Introduction

Mitochondria play a key role in apoptosis induction/execution. Specifically, the release of cytochrome *c* from the intermembrane space stimulates a caspase cascade that results in various morphological and biochemical manifestations of apoptosis. Mitochondria that release cytochrome *c* have lower membrane potentials and produce more reactive oxygen species (ROS) due to the leakage of electrons and the formation of superoxide radicals [1]. Elimination of these damaged mitochondria by mitophagy can improve cell survival but, at the same time, significantly attenuate apoptosis and hence diminish the effect of anticancer drugs [2]. Thus, mitophagy can play a role as a regulator of apoptosis.

Mitochondria are the major consumers of oxygen in the cell. Consequently, they are sensitive to a decrease in oxygen availability. One of the key strategies in response to hypoxia is the reduction of mitochondrial mass by mitophagy [3]. Mitophagy is mitochondria-targeted macroautophagy, an evolutionarily conserved mechanism. A double-membrane structure, called a phagophore, matures and sequestrates cellular components into a closed structure known as an autophagosome. The autophagosome fuses with the lysosome, where the cargo or the whole organelle can be utilized. Autophagy receptors provide selectivity for this process; they bind both cargo and mammalian ATG8 family proteins (LC3A, LC3B, LC3C, GABARAP, GABARAPL1, and GABARAPL2) attached to the phagophore [4]. Damaged mitochondria can be degraded by the PINK1-Parkin-Ubiquitin quality control system [5] or by receptor-mediated mitophagy in which several outer membrane proteins (BNIP3, BNIP3L, also known as NIX, FUNDC1, FKBP8, and Bcl2-L-13) interact with mammalian ATG8 family proteins [6,7,8,9]. A prerequisite for mitophagy is mitochondrial fission, which involves breaking down the mitochondrial network to individual organelles. Mitochondria are highly dynamic structures that fuse and divide, maintaining the tubular network. Under stress conditions, such as ROS stimulation or hypoxia, mitochondria start to divide. The key role in fission belongs to dynamin-related protein (Drp1), which oligomerizes on the mitochondria surface [10].

In addition to mitophagy stimulation, BNIP3 (Bcl-2 nineteen-kilodalton interacting protein 3) is a proapoptotic BH3-only protein. Like other Bcl-2 family members, BNIP3 binds to Bcl-2 and facilitates cytochrome *c* release through Bax/Bak-mediated pore formation in the outer mitochondrial membrane (OMM). In addition, BNIP3 can be localized to the endoplasmic reticulum (ER) [7]. Localization of BNIP3 to the ER membrane facilitates release of Ca^2+^ and subsequently its accumulation in mitochondria. Ca^2+^ loading can trigger opening of mitochondrial permeability transition pores with subsequent rupture of the OMM and decreased mitochondrial membrane potential, resulting in execution of caspase-independent cell death [11]. Thus, the role of BNIP3 in the regulation of cell death/survival is, to some extent, controversial.

Mitochondrial damage can stimulate not only mitophagy but also apoptotic cell death through the mitochondrial pathway. The targeting of complexes in the respiratory chain can significantly enhance apoptosis induced by antitumor drugs [12]. Mitophagy, through the elimination of damaged mitochondria, can modulate apoptotic pathways. To analyze how hypoxic conditions can affect mitochondrial quality control and modulate an interplay between apoptosis and mitophagy, we used thenoyltrifluoroacetone (TTFA) as an inhibitor of Complex II (succinate dehydrogenase) and combined this agent with the chemotherapeutic drug cisplatin in normoxic and hypoxic conditions.

## 2. Results

**Figure 1:**
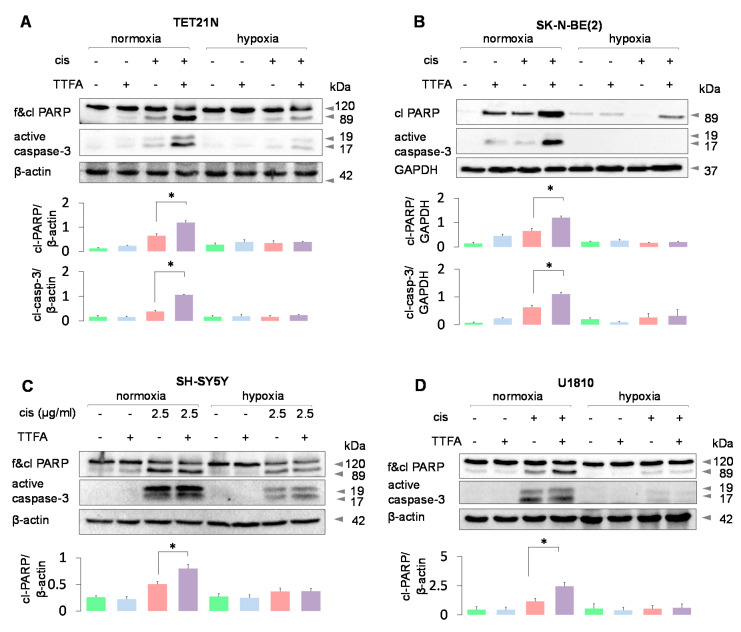
Hypoxia suppresses cell death induced by cisplatin in the presence or absence of TTFA, an inhibitor of Complex II of the mitochondrial respiratory chain. Assessment of apoptotic manifestations in Tet21N (**A**), SK-N-BE(2) (**B**), SH-SY5Y neuroblastoma cells (**C**), and lung large cell carcinoma U1810 cells(**D**). Bars under blots show quantification of Western blot data based on three independent experiments. * *p* < 0.01.

Under normoxic conditions, the effect of cisplatin, a conventionally used anticancer drug, is markedly increased in the presence of TTFA, an inhibitor of Complex II in the mitochondrial respiratory chain [12]. Indeed, in TET21N, SK-N-BE(2), and SH-SY5Y neuroblastoma cells (Figure 1A–C, respectively), as well as in human lung large cell carcinoma U1810 cells (Figure 1D), cotreatment with TTFA stimulated cisplatin-induced apoptosis. Cell death was assessed by the appearance of the processed form of caspase-3 (19-kDa and 17-kDa), and cleavage of poly (ADP-ribose) polymerase (PARP), a target of executive caspase-3, and the appearance of an 89-kDa cleavage fragment. Under hypoxic conditions, cisplatin-induced apoptosis was markedly suppressed in all cell lines, and almost no stimulation of apoptosis by TTFA was observed.

**Figure 2:**
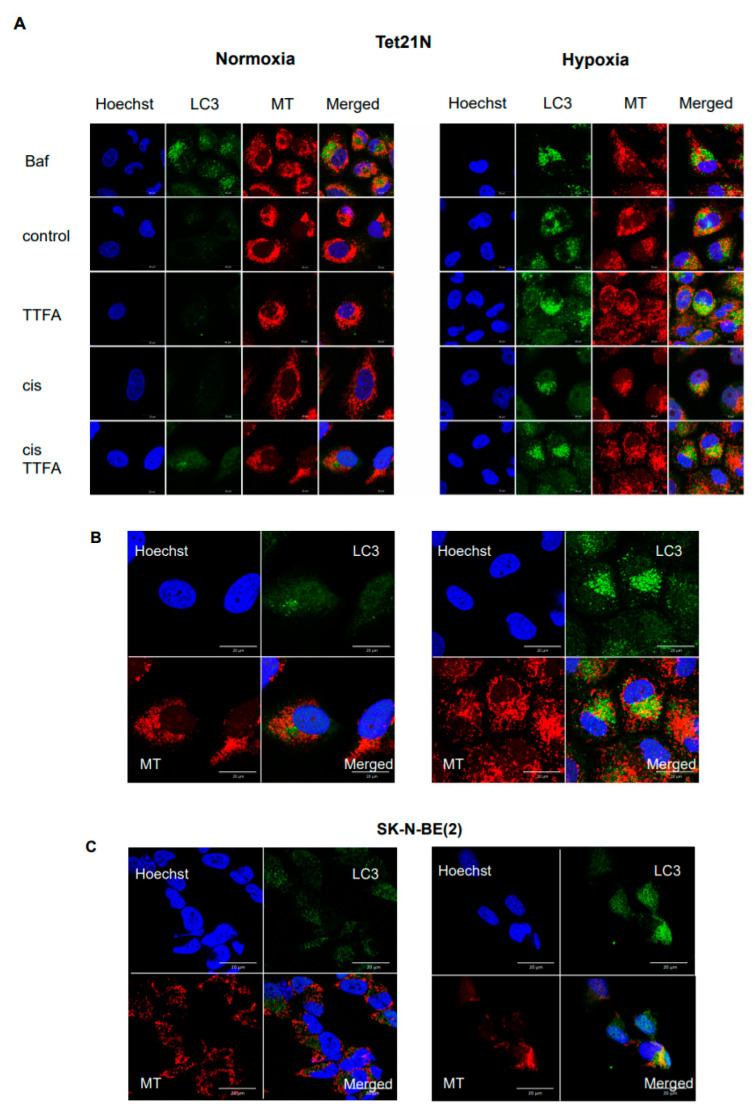
Hypoxia stimulates LC3 accumulation in Tet21N cells. Cells were stained with Hoechst (first column), antibodies against LC3II (second column), and mitochondrial dye Mitotracker CMXRos (third column). The fourth column represents merged images (**A**). Panels (**B**,**C**) represent high-magnification images of LC3 colocalization with mitochondria in Tet21N and SK-N-BE(2) cells treated with cisplatin and TTFA under normoxic and hypoxic conditions. Scale bar: 20 µm.

We previously showed that stimulation of mitophagy suppressed cisplatin-induced apoptosis, while mitophagy inhibition stimulated cell death [2]. In order to prove whether the attenuation of apoptosis under hypoxic conditions involves stimulation of mitophagy/autophagy, confocal images of cells were obtained after staining cells with Hoechst (first column), antibodies against LC3 (second column), and mitochondrial dye Mitotracker CMXRos (MT) (third column) (Figure 2); the fourth column represents the merged images. In these experiments, Bafilomycin A was used to inhibit degradation of autophagic cargos in lysosomes and to follow LC3 accumulation. Under normoxic conditions, accumulation of LC3 after treatment with Bafilomycin was observed in the cytosol and autophagosomes that were not significantly colocalized with mitochondria. In contrast, hypoxia caused colocalization of LC3 almost exclusively with mitochondria. This can be viewed as stimulation of the process of mitochondrial elimination. Panels B and C represent high-magnification images of LC3 colocalization with mitochondria in Tet21N and SK-N-BE(2) cells treated with cisplatin and TTFA under normoxic and hypoxic conditions.

As has been mentioned, mitophagy can be both PINK1/Parkin or receptor-mediated. A prerequisite step for triggering Parkin-mediated mitophagy is the stabilization of PINK1 at the OMM. In healthy mitochondria, PINK1 is imported through the OMM and partially through the inner mitochondrial membrane (IMM), followed by the cleavage of full-length PINK1, which is rapidly degraded in the proteasomes. In damaged mitochondria, PINK1 stabilizes on the OMM, phosphorylates other OMM proteins, and provides recruitment of Parkin. Activated Parkin conjugates ubiquitin chains to OMM proteins, which recruit autophagy receptors to eliminate mitochondria [5].

**Figure 3:**
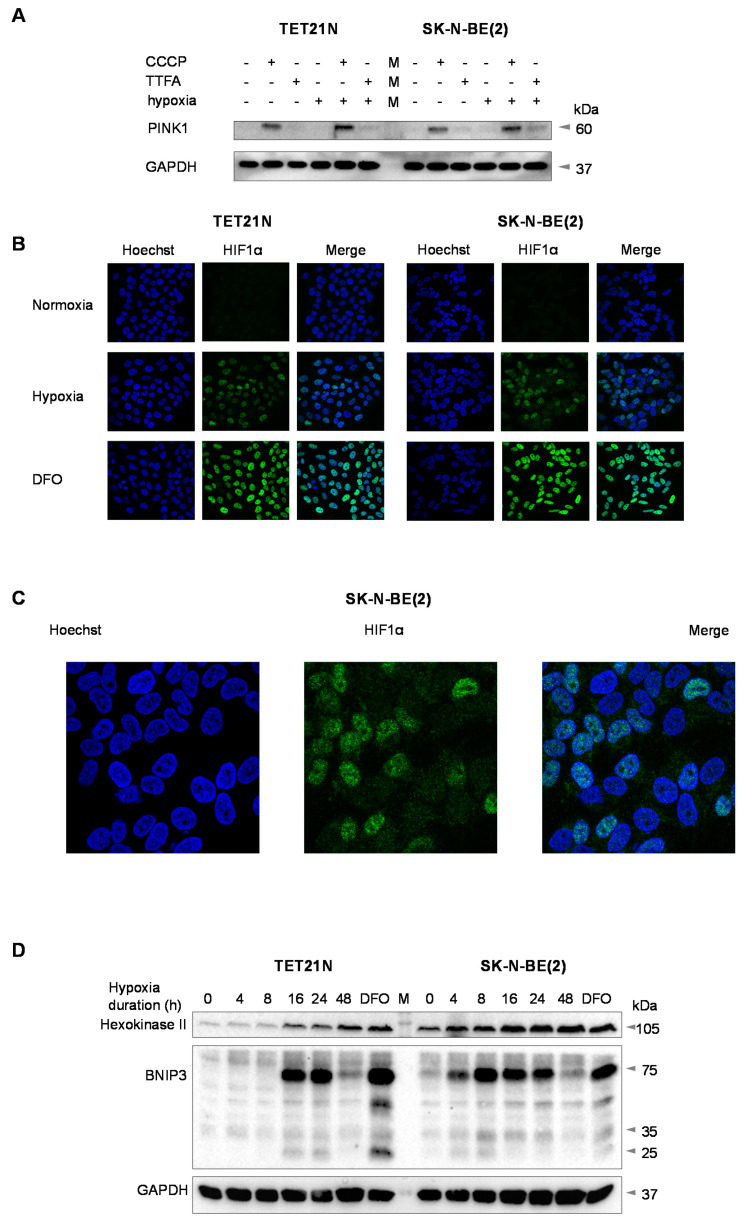
The consequences of hypoxia in Tet21N and SK-N-BE(2) neuroblastoma cells. Assessment of PINK1 accumulation (**A**), HIF1α stabilization and its colocalization with nucleus, stained with Hoechst (**B**), high-magnification images of HIF1α colocalization with nucleus in SK-N-BE(2) cells under hypoxic conditions (**C**), time-dependent accumulation of hexokinase II and BNIP (**D**). Deferoxamine (DFO) was used as a chemical mimetic of hypoxia, and GAPDH as a loading control. Scale bar: 20 µm.

To determine which pathway is triggered by hypoxia, mitochondria were treated with the protonophore carbonyl cyanide m-chlorophenylhydrazone (CCCP), which dissipates the mitochondrial membrane potential, or TTFA, and the level of PINK1 was assessed in Tet21N and SK-N-BE(2) neuroblastoma cell lines using gel electrophoresis and subsequent Western blotting. CCCP caused the appearance of PINK1 under both hypoxic and normoxic conditions; however, no PINK1 was detected upon stimulation of treatment with TTFA (Figure 3A). These data exclude the PINK1/Parkin-dependent pathway in mitophagy stimulation.

Hypoxia stabilizes HIF1α, a transcription factor, which activates the expression of a variety of genes. Incubation of TET21N and SK-N-BE(2) neuroblastoma cells under 0.1% O_2_ for 24 h induced HIF1α stabilization and its translocation into the nuclei (Figure 3B). Panel C represents high-magnification images of HIF1α colocalization with nucleus in SK-N-BE(2) cells under hypoxic conditions. Stabilization of HIF1α triggered the expression of various targets, among them hexokinase II, an enzyme that is upregulated under hypoxic conditions and stimulates glycolysis when mitochondrial ATP production is compromised [13]. The hexokinase II content was estimated after 4, 8, 16, 24 and 48 h of hypoxic incubation, and under normoxic conditions in the presence of 100 μM deferoxamine, an iron chelator, which is widely used to mimic hypoxic conditions. The stabilization of HIF1α also triggered the expression of the mitophagy receptor BCL2/adenovirus E1B 19 kDa protein-interacting protein 3 (BNIP3), which participates in cell death and mitophagy [14]. A similar result was observed in normoxia in the presence of deferoxamine (Figure 3D). The dimerization of BNIP3 on the mitochondrial outer membrane allowed its interaction with LC3B to engulf silent or damaged mitochondria [7]. The appearance of BNIP3 points to the involvement of receptor-mediated pathways in mitophagy initiation.

**Figure 4:**
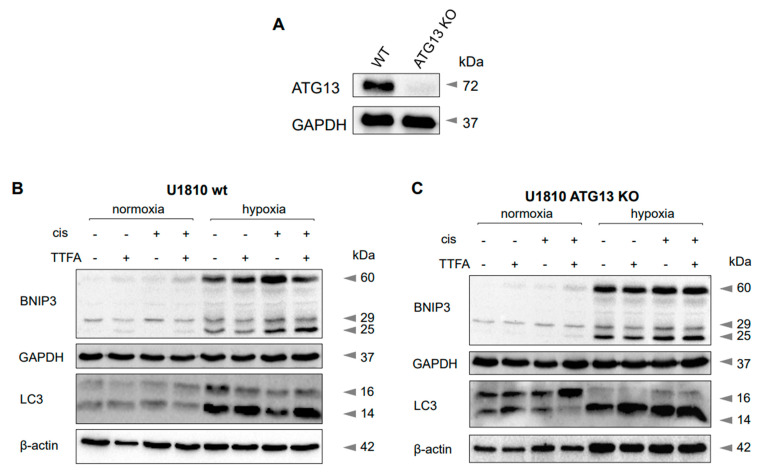

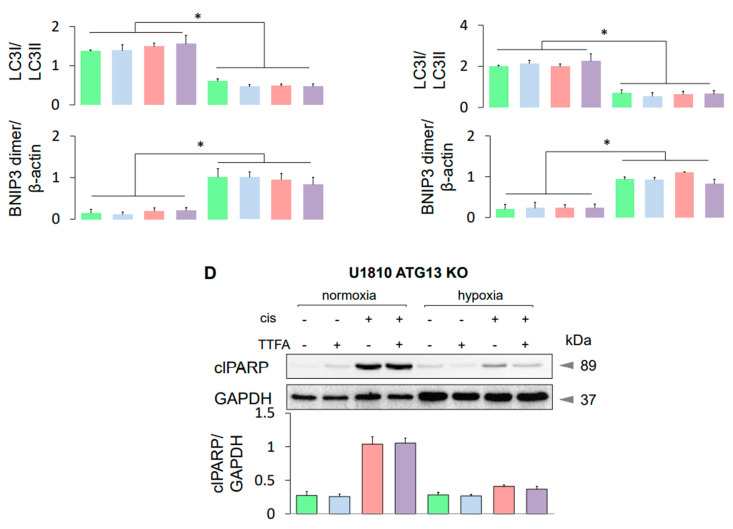
The consequences of hypoxia in wild-type (WT) and ATG13 knockout (KO) U1810 cells. The ATP13 content in WT and KO U1810 cells (**A**), hypoxia-triggered accumulation of BNIP3 and LC3 in WT (**B**) and ATG13 KO U1810 cells (**C**), hypoxia-induced suppression of apoptosis in ATP13 KO U1810 cells (**D**). GAPDH and β-actin were used as loading controls. Bars under blots show quantification of Western blot data based on three independent experiments; * *p* *<* 0.01.

To exclude the contribution of canonical macroautophagy in LC3II accumulation, U1810 cells lacking ATG13, a key member of the autophagy initiation complex, were used. A loss of ATG13 inhibits autophagy, and LC3-II levels failed to increase with the treatment of autophagy inducers [15]. Despite the absence of ATG13 in these cells (Figure 4A), the BNIP3 dimer and lipidated form of LC3B were still increased in hypoxia (Figure 4C). The obtained results confirm that when the canonical macroautophagy pathway is interrupted, mitophagy proceeds via a receptor-mediated pathway. Moreover, the absence of ATG13 did not affect the pro-survival role of mitophagy assessed by PARP cleavage (Figure 4D).

**Figure 5:**
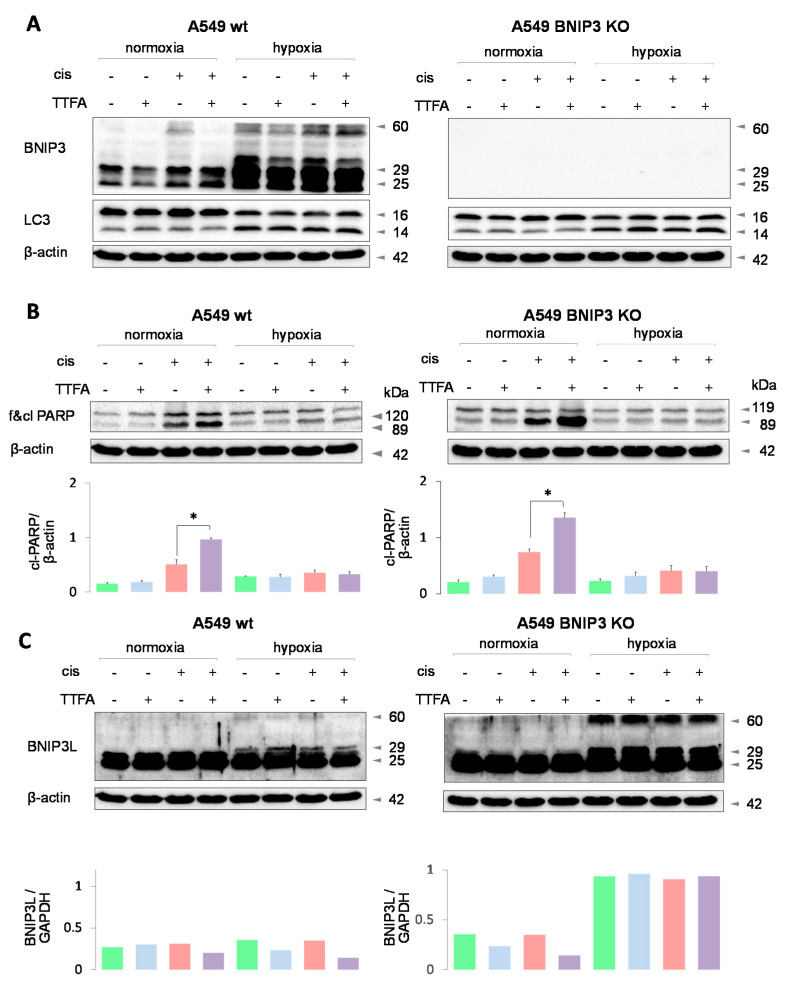
The consequences of BNIP3 knockout for cell death induction by cotreatment with cisplatin and TTFA under normoxic and hypoxic conditions in A549 lung adenocarcinoma cells. Assessment of LC3 content in WT and BNIP3 KO A549 cells (**A**), assessment of apoptotic PARP cleavage in WT and BNIP3 KO A549 cells (**B**), accumulation of BNIPL dimers in BNIP3 KO A549 cells (**C**). * *p* < 0.01.

To substantiate the protective role of BNIP3 in cisplatin-induced apoptosis, BNIP3-deficient lung adenocarcinoma A549 cells were used (Figure 5A). Surprisingly, a knockout of BNIP3 did not affect hypoxia-dependent protection against apoptosis as assessed by PARP cleavage (Figure 5B), which was observed in wild-type A549 cells. Moreover, the LC3 profile remained the same as in wild-type cells (Figure 5A). This brings into question the role of BNIP3 in mitophagy-mediated cell survival. However, it turned out that in BNIP3 knockout cells, hypoxia triggered the formation of BNIP3L dimers (60 kDa band; Figure 5C), which compensate for the lack of BNIP3 in cells with a BNIP3 knockout. In contrast to BNIP3, BNIP3L dimerization was not detected in wild-type A549 cells in hypoxia. Bars under blots show quantification of Western blot data. Thus, the survival of BNIP3 knockout cells in hypoxia can be explained by the compensatory formation of BNIP3L dimers and BNIP3L-mediated mitophagy stimulation.

**Figure 6:**
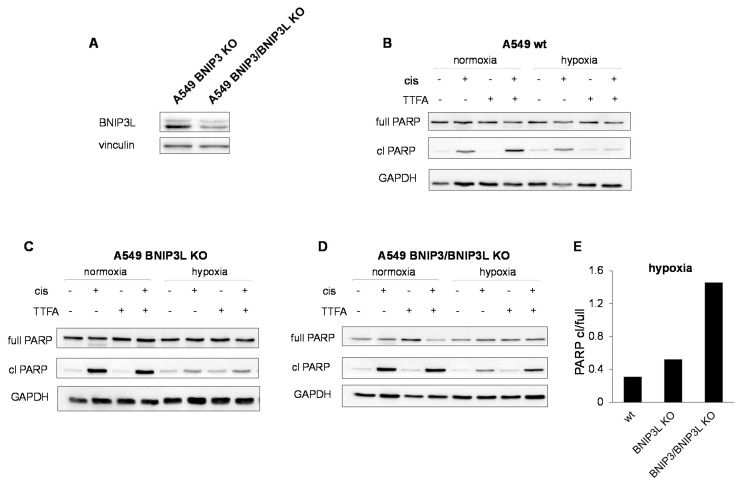
The role of BNIP3L in hypoxia-mediated suppression of apoptosis. The level of BNP3L in A549 cells lacking BNIP3 and in cells with BNIP3 double knock-out (**A**), assessment of PARP cleavage in wild-type (**B**), BNIP3L KO (**C**), BNIP3 and BNP3L double KO (**D**) A549 lung adenocarcinoma cells under hypoxic and normoxic conditions. (Panel **E**) represents the ratio of cleaved PARP to full length PAPR in cells treated with cisplatin and TTFA under hypoxic conditions.

To determine to what extent the compensatory upregulation of BNIP3L contributes to cell death suppression under hypoxic conditions, in A549 cells lacking BNIP3 the expression of BNIP3L was knocked out using the CRISPR/Cas9 system. Figure 6A represents the level of BNIP3L in A549 cells lacking BNIP3 and in cells with BNIP3 double knock-out. Panels B-D show apoptosis assessment by PARP cleavage in wild type A549 cells, BNIP3L KO cells, and cells lacking both BNIP3 and BNIP3L, respectively. In Figure 6E, the cleaved PARP to full PARP ratio is shown. As can be seen, in cells lacking both BNIP3 and BNIP3L, cleavage of PARP increased. This means that in the absence of both BNIP3 and BNIP3L suppression of apoptosis by hypoxia was reversed.

## 3. Discussion

Targeting mitochondria is an important tool in tumor cell elimination. Inhibition of the mitochondrial respiratory chain, which leads to the generation of ROS due to leakage of electrons, facilitates permeabilization of the mitochondrial outer membrane, a key event in triggering the mitochondrial apoptotic pathway. However, the elimination of damaged (proapoptotic) mitochondria by mitophagy can lessen therapeutic effects. As we have shown previously, stimulation of mitophagy in cells treated with the anticancer drug cisplatin attenuated cell death, whereas mitophagy inhibition stimulated apoptosis [2]. Thus, mitophagy-mediated elimination of permeabilized mitochondria and, hence, suppression of the mitochondrial apoptotic pathway, can contribute to tumor cell resistance to treatment.

Here we analyzed the mechanisms of tumor cell resistance in hypoxia. Under normoxic conditions, targeting mitochondria with TTFA, an inhibitor of Complex II in the respiratory chain, stimulated cisplatin-induced apoptosis in various cell lines, which was largely suppressed under hypoxic conditions (Figure 1A–D). This suppression could be due to the stimulation of canonical autophagy/mitophagy, which eliminates proapoptotic mitochondria (organelles generating ROS, depolarized, and with permeabilized OMM). However, under the conditions employed, there was no increase in the level of PINK1 (Figure 3A), a key component in canonical mitophagy induction. The increase in PINK1 content was detected only after the dissipation of the mitochondrial membrane potential caused by the uncoupler CCCP. These results prove that canonical mitophagy is not stimulated.

Hypoxic conditions stabilized HIF1α (Figure 3B) with subsequent activation of the expression of numerous genes involved in cellular metabolism reprogramming, in particular hexokinase II (Figure 3D), metastasis progression, and escape from programmed cell death [16]. In addition, HIF1α triggered the expression of the mitophagy receptor BNIP3 (Figure 3D). This protein can activate Parkin-independent mitophagy to eliminate damaged mitochondria that can trigger the intrinsic apoptosis pathway.

To prove that macroautophagy is not involved in apoptosis suppression under hypoxic conditions, experiments were conducted using U1810 cells lacking ATG13, a key enzyme of canonical autophagy (Figure 4A). ATG13 knockout did not affect the suppression of apoptosis (Figure 4D) and, at the same time, there was no alteration of the LC3 profile under hypoxic conditions. In both cell lines, hypoxia triggered the accumulation of BNIP3 dimers essential for the recruitment of autophagic machinery (Figure 4B). To prove that BNIP3 drives mitophagy in hypoxia-rescuing tumor cells, the experiments were carried out using BNIP3 knockout A549 cells. Surprisingly, the absence of BNIP3 did not affect either the LC3 profile (Figure 5A) or hypoxia-mediated apoptosis suppression (Figure 5B). At first glance, these results cast doubt on the involvement of BNIP3 in cell death suppression due to mitophagy induction. However, subsequent experiments revealed that although the expression of BNIP3 was successfully blocked in these cells, the expression of BNIP3L, which is also capable of mitophagy stimulation, was increased. The accumulation of BNIP3L dimers was shown to recruit autophagosomes more robustly than their monomeric form [17] (Figure 5C).

When both BNIP3 and BNIP3L were knocked out, the inhibitory effect of hypoxia on apoptosis was diminished, although not abolished completely. Undoubtedly, mitophagy is likely to be a mechanism responsible for cell death suppression, but not the mechanism. Indeed, we recently demonstrated that hypoxia could inhibit apoptosis upstream of mitochondria. We showed that under hypoxic conditions, suppression of p53 expression was responsible for the inhibition of apoptosis in HCT116 colon carcinoma and A549 lung adenocarcinoma cells through the downregulation of proapoptotic members of Bcl-2 family proteins [18].

**Figure 7:**
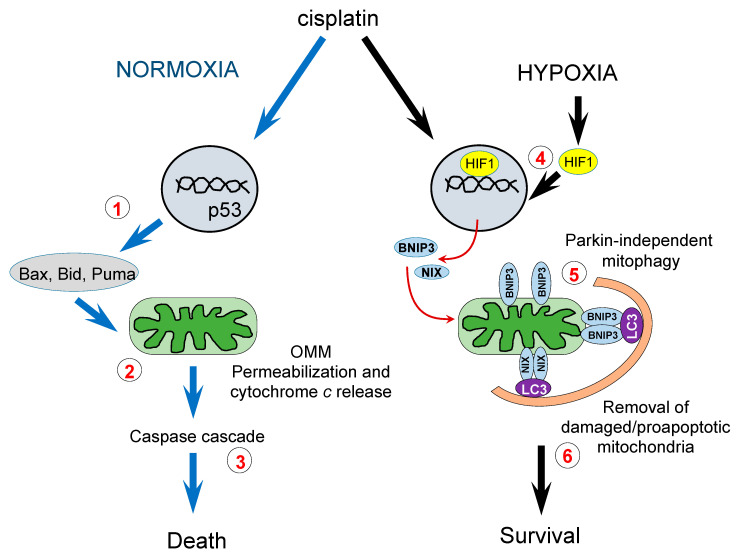
Cell survival under hypoxic conditions via activation of receptor-mediated mitophagy. See text for explanation.

The pathways of hypoxia-mediated cell death suppression are shown in Figure 7. In normoxic conditions, cisplatin stimulates apoptosis through DNA damage, which triggers the stabilization of p53, responsible for the expression of pro-apoptotic members of Bcl-2 family proteins (1). These proteins facilitate pore formation in the OMM and the release of cytochrome *c* from the intermembrane space (2) that triggers downstream caspase cascade leading to cell death (3). Hypoxia stabilizes HIF1α, which among other targets, stimulates expression of BNIP3 and BNIPL (4). The appearance of these proteins triggers receptor-mediated (Parkin-independent) mitophagy (5) and eliminates mitochondria with damaged membranes, which can be engaged in apoptosis stimulation. As a result, cell death is suppressed, and cells survive (6).

Thus, the obtained results demonstrate that under hypoxic conditions, BNIP3 and BNIP3L can rescue tumor cells treated with antitumor drugs by executing mitophagy. Our study showed that anticancer strategies should be chosen cautiously in order to prevent stimulation of survival pathways.

## 4. Materials and Methods

### 4.1. Cell Culture and Treatments

Studies were performed using human neuroblastoma SK-N-BE(2), SH-SY5Y, TET21N cells, and human lung large cell carcinoma U1810 cells and lung adenocarcinoma A549 cells. SK-N-BE(2) and SH-SY5Y cells were grown in complete DMEM (Waltham, Massachusetts, MA, United States) medium, containing 10% inactivated bovine serum (Gibco, Carlsbad, CA, USA), in the presence of antibiotics (penicillin, streptomycin, 100 U/mL) (Gibco) and sodium pyruvate (PanEco, Russia) in a CO_2_ incubator (5% CO_2_) (NuAire, Plymouth, MN, USA, nu-5500E) at 37 °C. U1810 and A549 cells were grown in complete RPMI 1640 (Thermo Fisher Scientific, Waltham, Massachusetts, MA, United States) medium. For TET21N cells, hygromycin (100 U/mL) and geneticin (200 U/mL) were added to the complete RPMI 1640 (Thermo Fisher Scientific, Waltham, Massachusetts, MA, United States) medium.

Hypoxia was established in a New Brunswick Galaxy 48R CO_2_ incubator (New Brunswick Scientific Co., Inc., Edison, NJ, USA), which allowed the maintenance of a 0.1% O_2_ level. Cells were incubated under normoxic or hypoxic conditions at 0.1% oxygen for 24 h, and after treatment incubated for the next 24 h under the same conditions, unless stated otherwise.

Cells were maintained at a logarithmic growth phase for all experiments. Apoptosis was induced by 5 µg/mL cisplatin (Ebewe Pharma, Unterach, Austria) for all cells in the presence or absence of 100 µM TTFA (Sigma-Aldrich, St. Louis, MO, USA, T27006). To block the final stage of autophagy, cells were treated with 100 nM bafilomycin A1 (Sigma-Aldrich, St. Louis, MO, USA, B1793).

### 4.2. Antibodies

Antibodies to the following proteins were used: anti-rabbit cleaved PARP (9541), anti-rabbit GAPDH (2118), anti-rabbit cleaved caspase-3 (9664), anti-rabbit PINK1 (D8G3), anti-rabbit hexokinase II (C64G5), anti-rabbit BNIP3 (D7U1T) (all from Cell Signaling Technology), anti-rabbit lc3b (ab51520), anti-mouse α-tubulin (ab176560), anti-rabbit β-actin (ab227387), anti-rabbit BNIP3L (ab8399) (all from Abcam).

### 4.3. Western Blotting

After 24 h, cells were harvested and dissolved in lysis buffer (100 mM Tris-HCl, pH 6.8, 4% SDS, 20% glycerin, and 200 mM dithiothreitol). Protein concentration was measured using the Pierce BCA Protein Assay Kit (Thermo Scientific, Waltham, MA, USA) according to the manufacturer’s instructions. Samples were mixed with Laemmle’s loading buffer and boiled for 7 min. Equal amounts (20–40 µg) of protein extracts were loaded to sodium dodecyl sulfate polyacrylamide gel electrophoresis (SDS-PAGE, 12% gel) at 100 mV, followed by blotting onto nitrocellulose membranes for 1 h 50 min at 110 V on ice. After transfer, nitrocellulose membranes stained with Ponceau S. Membranes were blocked for 1 h with 5% nonfat milk (AppliChemGmbH, Darmstadt, Germany, A0830) in tris-buffered saline (TBS) at room temperature, probed overnight with the appropriate primary antibodies (1:3000–1:5000) and followed by incubation with horseradish peroxidase-conjugated secondary antibodies (Cell Signaling Technology, anti-mouse 7076S or anti-rabbit 7074S, 1:4000) for 1 h with 2.5% nonfat milk in Tris-buffered saline (TBS) at room temperature. Detection was visualized with ECL (Amersham Biosciences, Piscataway, NJ, USA) and ChemiDoc MP Imaging System (BioRad). The quantitative analysis of Western blot data was accomplished using ImageJ Software (NIH, Bethesda, Rockville, MD, USA).

### 4.4. Immunocytochemistry

Cells were grown and treated on sterile glass coverslips as described above. Then cells were washed three times in Dulbecco’s phosphate-buffered saline (DPBS) and fixed in 4% paraformaldehyde in PBS pH 7.4 for 10 min at room temperature. After three washings, cells were permeabilized with DPBS containing 0.1% Triton X-100 for 15 min at room temperature. To block unspecific binding, cells were incubated with 1% BSA in PBST for 45 min. Cells were incubated in the diluted (according to the manufacturer’s instruction) primary antibodies in 1% BSA in PBST overnight at 4 °C. Cells were washed with PBST four times and incubated with diluted secondary antibodies (1:800 for mouse, 1:500 for rabbit) in 1% BSA in PBST in the dark and then washed three times with PBST. Nuclear DNA was labeled with Hoechst 33342 (in dilution 1:500) for 10 min. After three washings, coverslips were inverted onto a glass slip with a drop of mounting media (VECTASHIELD) containing a fluorescence antifade agent. Results were analyzed using a Zeiss LSM 510 META confocal laser scanner microscope (Carl Zeiss MicroImaging, Göttingen, Germany). The images obtained were quantified using ImageJ software.

### 4.5. Statistical Analysis

The results are presented as data from three independent experiments and expressed as the mean ± S.E.M (standard error of the mean). Statistical evaluation was performed using Mann-Whitney U-test. Values of *p* < 0.05 were considered significant.

## 5. Conclusions

Stimulation of cell death via targeting mitochondria is a promising strategy for tumor cell elimination. Under normoxic conditions, mitochondrial targeting successfully enhanced the anticancer effect of the widely used drug cisplatin. However, under conditions of hypoxia, the effect of a combined treatment significantly decreased. Tumor cells have developed mechanisms for survival and resistance to anticancer drugs. One such mechanism is the stimulation of mitophagy to eliminate mitochondria, which can trigger cell death. We found that the tumor cells were rescued by receptor-mediated mitophagy involving BNIP3 and BNIP3L proteins. When BNIP3 expression was blocked, mitophagy was stimulated by a compensatory expression of BNIP3L and accumulation of its dimers, which recruited autophagosomes more robustly than their monomeric form.

## Data Availability

Data is contained within this article.
